# Electrochromism of Viologen/Polymer Composite: From Gel to Insulating Bulk for High-Voltage Applications

**DOI:** 10.3390/ma14195901

**Published:** 2021-10-08

**Authors:** Yongjie Nie, Meng Zhang, Yuanwei Zhu, Yu Jing, Wenli Shi, Guoping Li, Haopeng Chen, Yihang Jiang, Xianping Zhao, Tengfei Zhao, Guanghao Lu, Shengtao Li

**Affiliations:** 1 Electric Power Research Institute, Yunnan Power Gird Co., Ltd., Kunming 650217, China; nieyongjie@stu.xjtu.edu.cn (Y.N.); zxpmy444@163.com (X.Z.); zhaotengfei@ncepu.edu.cn (T.Z.); 2 State Key Laboratory of Electrical Insulation and Power Equipment, and Frontier Institute of Science and Technology, Xi’an Jiaotong University, Xi’an 710049, China; zm0960@stu.xjtu.edu.cn (M.Z.); ligp16@xjtu.edu.cn (G.L.); chenhaopeng@stu.xjtu.edu.cn (H.C.); jyh0219@stu.xjtu.edu.cn (Y.J.); guanghao.lu@mail.xjtu.edu.cn (G.L.); sli@mail.xjtu.edu.cn (S.L.); 3 States Grid Shaanxi Economic Research Institute, Xi’an 710061, China; 18966890391@163.com; 4 Northwest Industrial Group Co., Ltd., Xi’an 710043, China; xbgybao@126.com

**Keywords:** insulation, polymer, space charge, high voltage, electrochromism

## Abstract

Power equipment operates under high voltages, inducing space charge accumulation on the surface of key insulating structures, which increases the risk of discharge/breakdown and the possibility of maintenance workers experiencing electric shock accidents. Hence, a visualized non-equipment space charge detection method is of great demand in the power industry. Typical electrochromic phenomenon is based on redox of the material, triggered by a voltage smaller than 5 V with a continuous current in μA~mA level, which is not applicable to high electric fields above 10^6^ V/m with pA~nA operation current in power equipment. Until now, no naked-eye observation technique has been realized for space charge detection to ensure the operation of power systems as well as the safety of maintenance workers. In this work, a viologen/poly(vinylidene fluoride-co-hexafluoropropylene)(P(VDF–HFP)) composite is investigated from gel to insulating bulk configurations to achieve high-voltage electrical-insulating electrochromism. The results show that viologen/P(VDF–HFP) composite bulk can withstand high electric fields at the 10^7^ V/m level, and its electrochromism is triggered by space charges. This electrochromism phenomenon can be visually extended by increasing viologen content towards 5 wt.% and shows a positive response to voltage amplitude and application duration. As viologen/P(VDF–HFP) composite bulk exhibits a typical electrical insulating performance, it could be attached to the surface of insulating structures or clamped between metal and insulating materials as a space charge accumulation indicator in high-voltage power equipment.

## 1. Introduction

Power equipment is the key factor for the safe and reliable operation of power systems [1,2,3]. As the economy rapidly grows, power load is synchronously increased dramatically. To solve the problem of regional power shortages, large-area ultra-high-voltage (UHV) power transmission is applied, in which power equipment operates under long-term extreme voltages [4,5,6]. As a consequence, the risk of insulating failure caused by discharge and breakdown is increased [7,8,9,10].

Polymers are the basic electrical insulating material applied in power equipment, and include: polyethylene (PE), which serves as the core material of power cables; polypropylene (PP), which serves as as the basic energy storage material in power capacitors; and epoxy resin (EP), which serves as the insulating support in gas-insulated switchgears (GIS) [11,12,13]. A common problem is that, under high voltages, space charges are formed through thermal emission and the tunneling effect, which accumulate on the surface of and inside the insulating polymer. These excessive space charges cannot dissipate as the high voltage is continuously stressed, and may form a local charge concentration, which generates a strong distorted electric field, consequently leading to partial discharge, and even breakdown [14,15,16,17]. Thus, discovering the accumulation of space charges at an early stage is vital for ensuring the stable operation of power equipment [18].

With regard to substation overhaul in the power industry, space charges that accumulate on key insulating structures may cause serious loss of human life. Substation operators and maintenance personnel will inevitably come into contact with insulating material to conduct condition checks. However, space charges can be residual on an insulator’s surface in the hours after power shutdown, increasing the possibility of an electric shock accident. Commonly, an electricity test is conducted before maintenance; however, this procedure is complicated, with large equipment involved. Thus, if the charged state of insulating materials could be visualized, it would be a direct signal warning of potential dangers. This non-equipment strategy could largely reduce costs and improve the comfort of inspectors, which exhibits the potential of large-scale application in the power industry.

Electrochromism refers to the phenomenon by which the color of a material varies in response to an electric signal, which exhibits the properties of observable and reversible color changes along with spectral signals [19,20]. Organic electrochromism is an emerging technology owing to its rich color changes, excellent flexibility, and solution processability in comparison with inorganic electrochromism materials [21,22]. Viologen is one of the most representative organic electrochromic materials [23,24,25,26], first discovered in 1973 [21]. So far, viologen has been widely used in the fields of electrochromic smart windows [22,27], auto anti-glare rearview mirrors [23], energy-saving displays [24], and multiple stimulus-responsive smart materials [28,29].

In the field of viologen-based electrochromism, the community is continuously looking for devices that operate with low voltage and low power consumption. In 2014, Xu et al. deposited TiO_2_ onto an electrode surface with viologen involved, and the ion diffusion was greatly increased, leading to a significantly improved discoloration speed, as well as an operation voltage of 1.8 V [30]. In 2015, a phosphor-containing viologen derivative was synthesized by Walter et al., and an ultra-low application voltage of 0.1 V was achieved [31]. In 2019, Li et al. introduced polynuclear–metal–carboxylic–acid clusters into viologen hybrid materials, which exhibited quick responses to soft X-ray, ultraviolet light, temperature, and electric fields under −0.6 V voltage [28]. In 2021, He et al. synthesized bismuth-containing viologen derivatives, which were successfully applied to electrophosphorescent devices with a working voltage of −0.6 V [25].

As summarized above, current electrochromism is based on low voltage (<5 V) [19,25,26,30,31], with a sustainable μA~mA level current to trigger the redox reaction. Thus, electrolytes and ionic liquids are usually added to improve conductivity and reduce the driving voltage for discoloration, which are considered contrary to the nature of insulating materials working under high electric fields above 10^6^ V/m with tiny currents at pA~nA level. Thus, current electrochromism structures are not applicable in power systems requiring electrical insulating conditions.

In this work, in order to broaden the applications of electrochromic materials to high-voltage conditions, a viologen/P(VDF–HFP) composite is systematically investigated from gel with semiconducting characteristics to bulk its electrical insulating characteristics.

## 2. Materials and Methods

**Figure 1:**
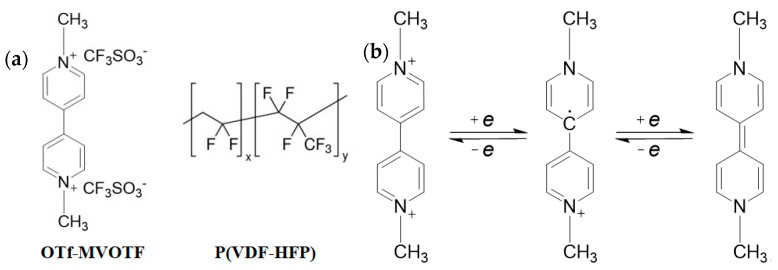
(**a**) Molecular structures of OTf–MVOTF and/P(VDF–HFP); and (**b**) electrochromic redox process of viologen (OTf–MVOTF).

**Materials.** The viologen used in this investigation is 1,1′-dimethyl-4,4′-bipyridine (OTf–MVOTF), a typical viologen small molecule. The polymer matrix is poly(vinylidene fluoride-co-hexafluoropropylene), which is shortened to P(VDF–HFP). The viologen was laboratory synthesized and the P(VDF–HFP) was purchased from Shanghai Huayi 3F New Materials Co., Ltd., China, and its *M*_w_ = 200 kDa. Figure 1a shows the molecular structures of the materials mentioned above. The electrochromic mechanism of OTf–MVOTF is shown in Figure 1b, with different valence states of OTf–MVOTF exhibiting diverse colors. The divalent cations are stable in a colorless state, the monovalent cations are blue, and the neutral particles show a blue-green color.

**Figure 2:**
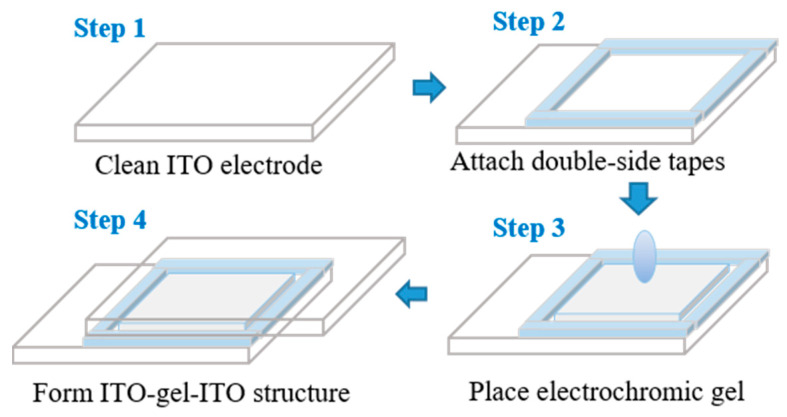
Structure and preparation of the viologen/P(VDF–HFP) electrochromic device.

**Electrochromic gel and devices fabrication.** Electrochromic composite gel with different mass ratios of viologen was prepared, viologen: P(VDF–HFP) = 1:99, 2:98, 3:97, 4:96, 5:95. In preparation, the P(VDF–HFP) and viologen at each content ratio were blended with 10 mL acetone in a beaker, heated, and stirred at 50 °C on a heater to form a uniformly mixed solution. With the volatilization of acetone, the solution gradually became a colorless and transparent gel until viscosity reached ~22 Pa·s (shear rate ɣ = 1/s) at room temperature, and the concentration of viologen/P(VDF–HFP) mixture was ~0.3 g/mL. The electrochromic device was composed of a transparent electrode and electrochromic gel. The electrochromic electrode applied was an ITO conductive glass with a transmittance greater than 84%, and the resistance of the electrode was 10 Ω/sq. In device fabrication, transparent double-side electrical insulating tape (width: 4 mm, thickness: 0.5 mm) was attached to one ITO-coated glass to form a cavity to fill with the electrochromic gel, and then another ITO-coated glass was placed on top of the gel to form the ITO/electrochromic gel/ITO structure. It is worth noting that the side with ITO was attached to the electrochromic gel. The preparation process of the electrochromic gel device is demonstrated in Figure 2.

**Viologen/P(VDF–HFP) composite bulk preparation.** The electrochromic gel mentioned above was placed in an open mold at room temperature. After two days, the solvent was completely volatilized, and the viologen/P(VDF–HFP) composite bulk without solvent was thus obtained.

**Characterization.** UV-Vis spectrometer was applied in characterizing the electrochromic performance and color change of the sample, and the test wavelength range was 350 nm~800 nm. A voltmeter was used to control the amplitude of the applied voltage. To trigger the electrochromism of viologen/P(VDF–HFP) insulating bulk, a space charge injection was conducted with a copper plate-plate electrode clamped onto the sample. A DC voltage of 6 kV (4 kV/mm) was applied to the viologen/P(VDF–HFP) bulk for up to 1 hour. Dielectric parameters such as dielectric constant and volume resistivity was obtained from Novocontrol Concept 80 (Germany). The applied signal was 1 V AC voltage, the test frequency range was from 10^−1^ to 10^6^ Hz, and the temperature range was from −100 to 100 °C. When testing, the insulating bulk was clamped by plate-plate copper electrodes. Cyclic voltammetry (CV) was tested by the CHI660E electrochemical workstation.

## 3. Results and Discussion

In this section, the electrochromism of viologen is realized in two configurations, i.e., the viologen/P(VDF–HFP) gel with semiconducting characteristics and viologen/P(VDF–HFP) bulk with electric insulating characteristics. The dielectric and insulating performance of the viologen/P(VDF–HFP) insulating bulk is further investigated to meet the application conditions of high-voltage power equipment.

**Figure 3:**
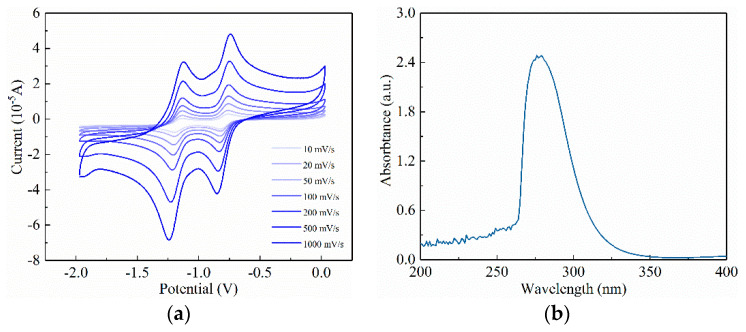
(**a**) The cyclic voltammogram of viologen at different scan rates in DMF solution; (**b**) the UV–Vis absorption of viologen in DMF solution, c = 10^−4^ mol/L.

The electrochemical properties of OTf–MVOTF were first investigated through cyclic voltammetry (CV) at different scan rates, the results of which are demonstrated in Figure 3a. The CV of viologen exhibits two reversible one-electron processes. Corresponding to these two valences, their reduction potentials are −0.72 V for monovalent cation and −1.10 V for neutral state, measured at the scan rate of 100 mV/s. Thus, the LUMO of viologen is calculated as −4.08 eV. In order to obtain the energy structure of viologen, UV-Vis spectrometry is conducted, and the results are shown in Figure 3b. The energy gap was calculated by *E*_g_ = 1240/λ_g_, where *E*_g_ is the energy gap and λ_g_ is the intersection of the tangent of the UV-Vis result and *x*-axis. The calculated energy gap of viologen is 3.97 eV, leading to its HOMO at −8.05 eV. These parameters related to the energy structure of viologen are summarized in Table 1.

### 3.1. Electrochromism of Viologen/P(VDF–HFP) Gel

#### 3.1.1. Electrochromism under 3 V Small Voltage

**Figure 4:**
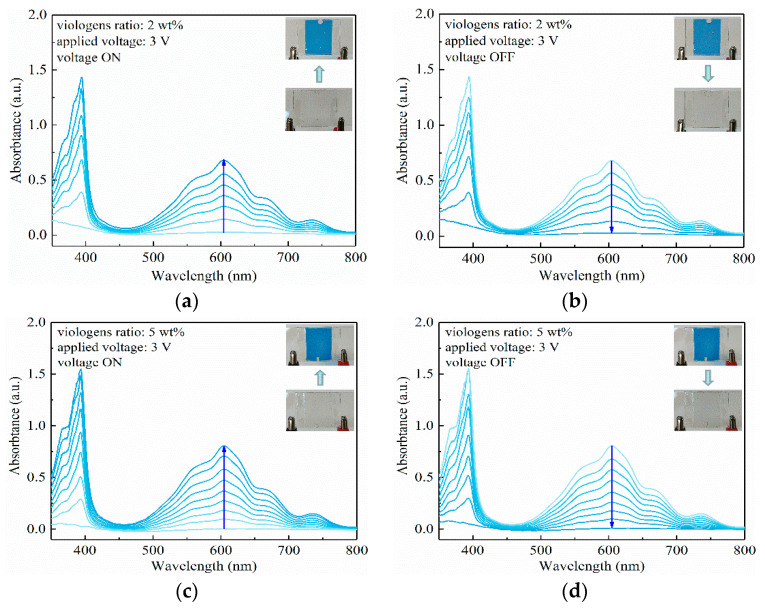
UV–Vis absorption characteristics of viologen (2 wt%)/P(VDF–HFP) gel with 3 V voltage ON (**a**) and OFF (**b**); UV–Vis absorption characteristics of viologen (5 wt%)/P(VDF–HFP) gel with 3 V voltage ON (**c**) and OFF (**d**).

The electrochromism performance of viologen/P(VDF–HFP) gel with 2 wt% viologen content was investigated first. The divalent cation state of viologen was transparent. With the application of 3 V voltage, the composite gel immediately turned to azure. In the UV–Vis absorption spectrum, the color change is expressed as the raise of peaks at 392 nm and 604 nm. It can be observed in Figure 4a that no absorption peak of viologen/P(VDF–HFP) gel can be seen without the applied voltage. As the color turned to azure, the peaks in 392 nm and 604 nm continued to increase, reaching 1.4 and 0.7 in absorptance, correspondingly. When the applied voltage was shut down, the gel turned colorless within 30 s, with the absorption peaks disappearing in Figure 4b, showing the quick response performance of the electrochromism composite. In terms of the mechanism, viologen gains electrons through a continuous current in μA~mA level, which triggers a reduction in the divalent cation to a monovalent cation, and contrarily, the viologen is oxidized back to a divalent cation when the voltage is shut down.

The electrochromism performance of the viologen/P(VDF–HFP) gel with 5 wt% viologen content under 3 V voltage is shown in Figure 4c,d. In terms of color change with the viologen content, the gel turned to darker azure in comparison to the gel with 2 wt% viologen content. Synchronously, the absorption peak at 392 nm is elevated from 1.4 to 1.6, which further indicates that the electrochromism is more violent. As more viologen molecules are involved in electrochromism, the color change is more obvious for visual observation. Another influencing factor is the electric conduction: since viologen is a typical semiconducting molecule, an increase in viologen content in a viologen/insulating polymer composite leads to decreased electric resistivity of the system. In other words, the density of mobile charges is increased, resulting in an easier process of ion exchange and redox. Consequently, the color changes to darker azure as a stronger electrochromism is triggered.

#### 3.1.2. Electrochromism under 5 V Large Voltage

**Figure 5:**
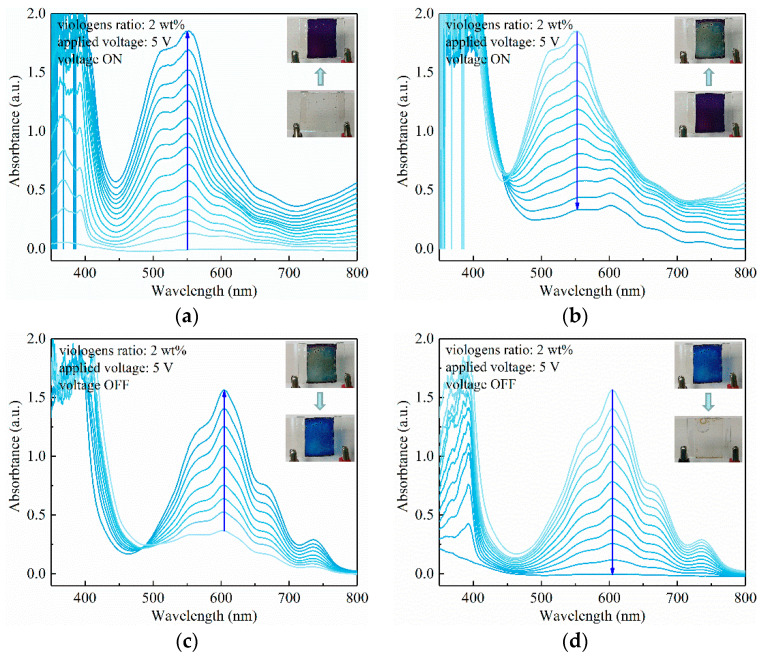
UV-Vis absorption characteristics of viologen (2 wt%)/P(VDF–HFP) gel with 5 V voltage ON (**a**,**b**) and OFF (**c**,**d**).

In testing the electrochromism of the viologen/P(VDF–HFP) gel system, it is apparent that increased applied voltage leads to a more complicated electrochromism process, as demonstrated in Figure 5.

In Figure 5a, it is observed that two electrochromism stages occur in the viologen/P(VDF–HFP) gel under 5 V voltage. Firstly, the colorless film turned purple in ~1 s with the absorption peaks at 392 nm and 555 nm, synergistically increasing. It should be noted that it is 604 nm when under 3 V small voltage, and this blue shift leads a coloration closer to purple. As the 5 V voltage is stably applied, the purple film turns to a blue-green color, with the 555 nm absorption peak decreasing while the 392 nm peak remains nearly unchanged, as shown in Figure 5b. This two-stage electrochromism phenomenon was induced by the two valences of viologen, which is azure in monovalent cation (+1 valence) and blue-green in neutral state (0 valence). In correspondence to these two valences, their related reduction potentials were −0.72 V for monovalent cation and −1.10 V for neutral state.

The reduction potential obtained by cyclic voltammetry is usually smaller than that of the viologen/P(VDF–HFP) gel composite, as it is obtained in solution with solely viologen as solute, the composite with polymer leads to an increased reduction potential. Therefore, it is reasonable to consider the neutral state reduction potential of viologen/P(VDF–HFP) gel composite is between 3 V and 5 V; thus, the applied 3 V could only trigger the viologen in composite form to monovalent cation.

In Figure 5c,d, the bleaching process is the opposite of the coloring process, as the film first turns azure with an elevation of a 604 nm peak, followed by a co-descent process of 392 nm and 604 nm peaks, and finally the film turns colorless as the oxidation process is completed. Interestingly, the 555 nm peak during the coloring process does not strictly correspond to the peak during bleaching at 604 nm, which needs further investigation.

**Figure 6:**
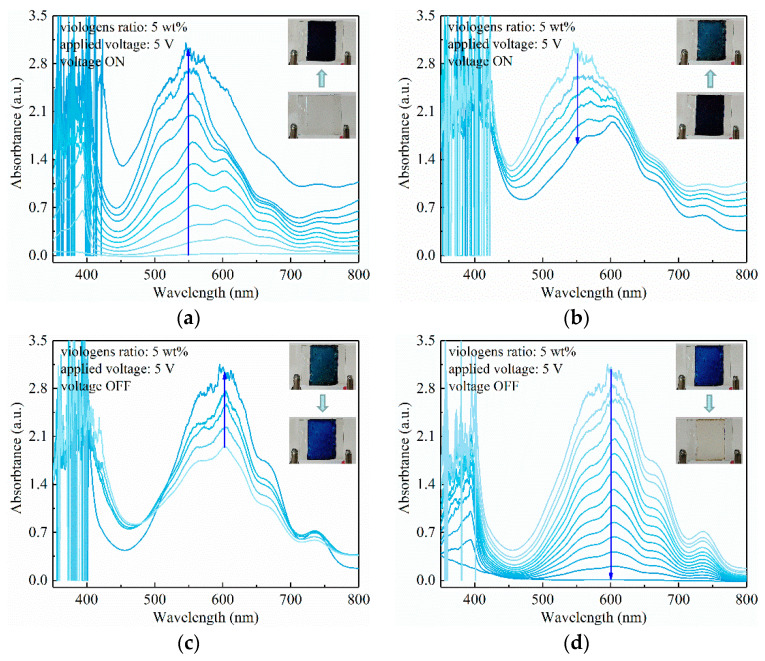
UV–vis absorption characteristics of viologen (5 wt%)/P(VDF–HFP) gel with 5 V voltage ON (**a**,**b**) and OFF (**c**,**d**).

In comparison to the 2 wt% viologen/P(VDF–HFP) gel, by increasing the content of viologen to 5 wt%, the electrochromism is further enhanced under 5 V voltage, as demonstrated in Figure 6. In Figure 6a,b, the coloring process exhibits two stages, which is in accordance with that of the 2 wt% viologen content gel in Figure 5. However, the coloring is more extensive as the deeper purple is realized, owing to the comparatively larger viologen density. The film could finally turn back to colorless as the applied voltage is shut down, showing the quick response of the electrochromism gel, as demonstrated in Figure 6c,d.

Generally, the electrochromism characteristics of viologen/P(VDF–HFP) gel with varied viologen content are investigated. Typical phenomena can be concluded as: (1) reversible electrochromism with a quick response is realized, in both coloring and bleaching processes; (2) the electrochromism can be triggered by a continuous current with a range of 3–5 V voltages; (3) two-stage electrochromism with a neutral state viologen can be realized with 5 V voltage, which is closely related to the reduction potential.

### 3.2. Electrochromism of Viologen/P(VDF–HFP) Insulating Bulk

#### 3.2.1. Electrochromism Characterization

**Figure 7:**
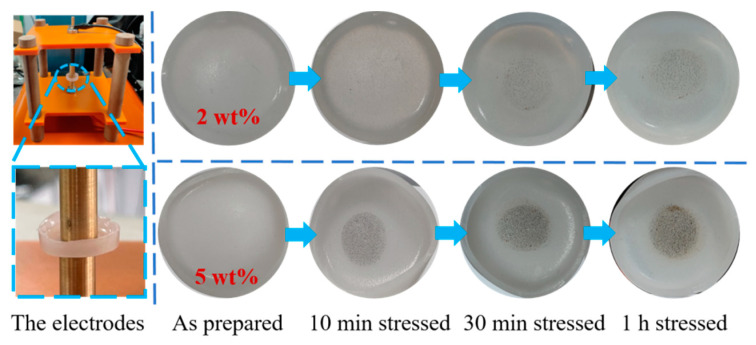
Electrochromism of 2 wt% and 5 wt% viologen/P(VDF–HFP)-insulating bulks under 6 kV voltages in up to 1 h.

The electrochromism of viologen/P(VDF–HFP) gel is triggered by a μA~mA level current, induced by 3–5 V voltages. However, power electrical equipment exhibits extreme high voltages in kV levels and electric fields greater than 10^6^ V/m, which can hardly be sustained by viologen/P(VDF–HFP) gel. Therefore, in order to introduce electrochromic materials for high-voltage applications, a viologen/P(VDF–HFP) composite is further prepared as insulating bulk. For the viologen/P(VDF–HFP) gel structure, ITO transparent electrodes are applied to transfer a continuous small current. As ITO glass cannot withstand high voltages, copper plate electrodes are introduced in a high-voltage measurement, as demonstrated in Figure 7.

A viologen/P(VDF–HFP) insulating bulk with 1~5 wt% viologen and a thickness of ~1.5 mm was prepared. Moreover, 2 wt% and 5 wt% viologen/P(VDF–HFP)-insulating composite bulks show a similar exterior to a polymer-like surface with a milky white color. A small content of viologen does not affect the transparency of the bulk, as it is mainly dependent on the condensed matter structure of the P(VDF–HFP) matrix.

In order to trigger the redox of viologen, 6 kV high voltage (4 kV/mm) is applied to the viologen/P(VDF–HFP)-insulating bulk for up to 1 h, and the results are demonstrated in Figure 7. For 2 wt% viologen/P(VDF–HFP)-insulating bulk, a slight but no significant color change is observed after 10 min stress. For 30 min stress using 2 wt% viologen/P(VDF–HFP)-insulating bulk, gray dots appear on center of the bulk in the area covered by the copper electrode. Since the voltage stressing duration is longer, the gray color appears more obvious on 1 h stressed sample. 

In Figure 7, the color change is more obvious on a 5 wt% viologen/P(VDF–HFP) insulating bulk at each voltage application duration. Contrary to 2 wt% viologen/P(VDF–HFP) bulk, 5 wt% viologen/P(VDF–HFP) bulk shows obvious gray dots after 10 min of stress, and this color change is even more obvious than 2 wt% viologen/P(VDF–HFP) bulk after 1 h of stress, indicating that the quantity of viologen involved in electrochromism is larger. By increasing the voltage application duration, the extent of the color change is extended, which increases the visualization of electrochromism. For the sample stressed for 1 h, the composite bulk changes to a dark gray, indicating that a large amount of space charges are injected, and triggering stronger electrochromism.

Thus, electrochromism is initially realized in the insulating polymer system. However, this electrochromism is not as obvious as that in the gel system, since the color change only occurs in the positions covered by electrodes, and the peripheral positions remain their intrinsic color. Regarding response duration, the insulating bulk exhibits more time in minute level required to trigger observable electrochromism, while that in the gel system only needs ~1 s. These differences in electrochromism resolution and duration are mainly caused by the number of charges required to trigger redox. In the gel system, a continuous μA-mA current provides sufficient charges for redox, while the electrical insulating system exhibits extremely small quantity of charges, which are insufficient for observable electrochromism when voltage is applied.

However, under extreme high voltages, charges can be emitted from metal electrodes through Schottky thermionic emission [32,33]. Commonly, accumulated space charges can be of a density of 10^19^ C/cm^3^, which is the minimum intensity required for electrochromism of viologen. As the high voltage is stressed, space charge density on the surface of the composite continues to grow. With more charges involved in the redox of viologen, the electrochromism becomes observable.

It should be noted that space charge accumulation usually exhibits a long duration towards the hour mark in order to achieve higher density; thus, electrochromism of viologen/P(VDF–HFP)-insulating bulk requires a longer duration for visual detection. As the electrochromism extent of viologen/P(VDF–HFP) bulks is in positive correlation with space charge density, it is an emerging method to quantitatively analyze the accumulated space charges in polymeric insulators; this is helpful in the detection of localized electric field distortions.

#### 3.2.2. Dielectric Characterization

In order to verify the application possibility of viologen/P(VDF–HFP) electrochromism bulk in electrical equipment, the dielectric and insulating performance of the composite bulk was further investigated.

**Figure 8:**
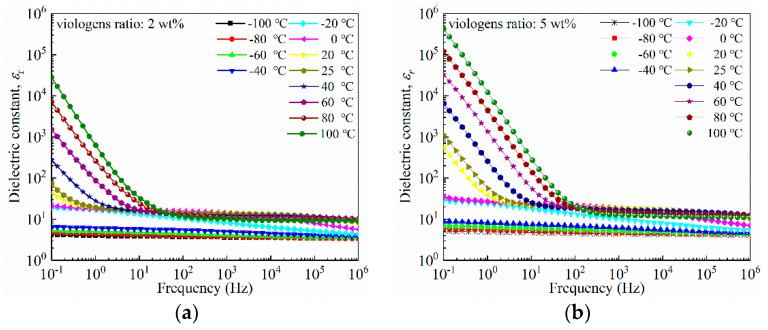
Dielectric spectra of 2 wt% (**a**) and 5 wt% (**b**) viologen/P(VDF–HFP) bulks in frequency of 10^−1^~10^6^ Hz, temperature of −100~100 °C.

As demonstrated in Figure 8a, the dielectric constant of viologen/P(VDF–HFP) bulk with 2 wt% viologen is 44 at room temperature (20 °C). In higher temperatures, the low frequency dielectric constant starts to elevate and gradually increases with increased temperature. At 100 °C, the dielectric constant is greatly enlarged, indicating board dielectric relaxation. As the temperature is above the glass transition temperature, the rotation of the polymer chain is simpler, as well as electric conduction of the semiconducting viologen being greatly enhanced, leading to enlargement in the dielectric constant. However, as the power frequency (50~60 Hz) dielectric constant is 14.4 and similar to that of P(VDF) (~10), viologen/P(VDF–HFP) bulk can be applied in insulating structures of power equipment [34].

A further increase in viologen content leads to an increase in the dielectric constant at all frequencies. As demonstrated in Figure 8b, the 50 Hz room temperature dielectric constant of viologen/P(VDF–HFP) bulk with 5 wt% viologen content is 20.1, showing a 39.5% increase compared to that of the 2 wt% viologen bulk. A further increase in viologen leads to a decrease in thermal stability, as *ε*_r_ starts to raise at a relatively lower temperature. Thus, viologen/P(VDF–HFP) bulk may not be applied to power equipment under elevated temperatures at this stage, as too much dielectric loss may be generated.

**Figure 9:**
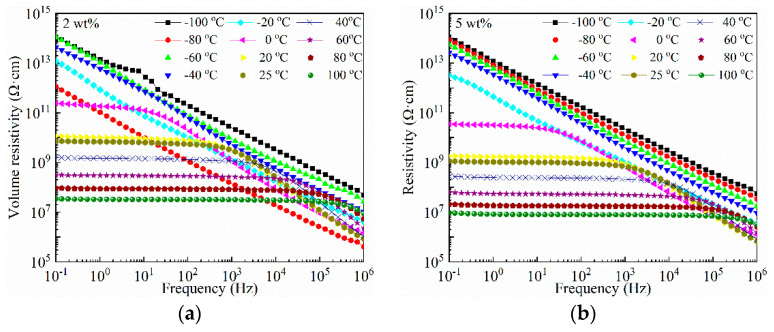
Volume resistivity spectra of 2 wt% (**a**) and 5 wt% (**b**) viologen/P(VDF–HFP) bulks in frequency of 10^−1^~10^6^ Hz, temperature of −100~100 °C.

The withstand voltage is greatly dependent on the resistivity of a dielectric polymer. For viologen, its volume resistivity is in a 10^7^~10^8^ Ω·cm level [35]. Since 10^8^ Ω·cm is the boundary between semiconductors and insulators, it is in the range of semiconductors. The volume resistivity of viologen/P(VDF–HFP) bulk is investigated, the results of which are demonstrated in Figure 9. In Figure 9a, the volume resistivity of viologen/P(VDF–HFP) bulk with 2 wt% viologen is in a 10^10^~10^11^ Ω·cm level at room temperature at frequencies below 10^3^ Hz, while it linearly decreases with increased frequency greater than 10^3^ Hz. Thus, viologen/P(VDF–HFP) composite bulk exhibits fundamental electrical insulating performance. A quasi-platform can be found in temperatures above 0 °C. For 5 wt% viologen/P(VDF–HFP) bulk in Figure 9b, the room temperature volume resistivity is decreased to 10^9^ Ω·cm; this is a result of increased semiconducting viologen content.

**Figure 10:**
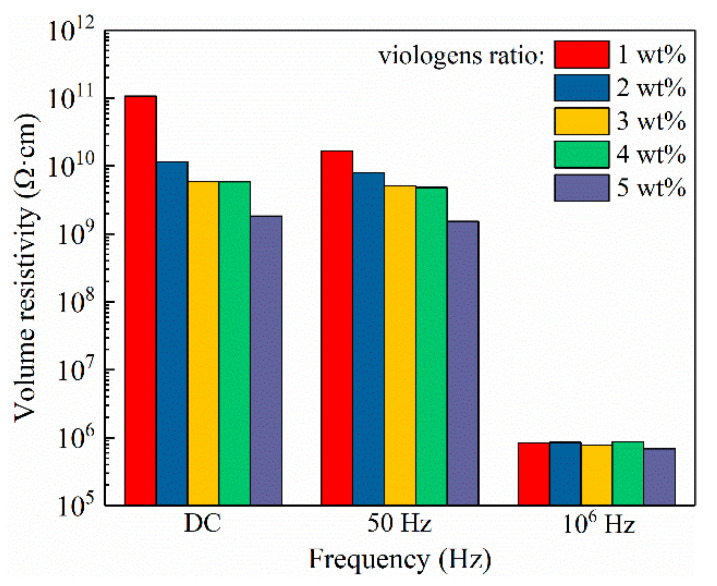
Variations in volume resistivity with 1 wt%~5 wt% viologen content in viologen/P(VDF–HFP) bulks.

At elevated temperatures, the volume resistivity of 5 wt% viologen/P(VDF–HFP) bulk is further decreased compared to that of the 2 wt% composite bulk. The variation trend of volume resistivity with viologen content is demonstrated in Figure 10. It is clearly demonstrated that DC volume resistivity of the composite bulk is the highest, followed by 50 Hz AC resistivity and 10^6^ Hz AC resistivity, respectively. The volume resistivity of viologen/P(VDF–HFP) bulk decreases with increased viologen content. For DC resistivity, the 1 wt% sample is 10^11^ Ω·cm, followed by the 2 wt% sample at 10^10^ Ω·cm, the 3 wt% sample at 5.9 × 10^9^ Ω·cm, and the 5 wt% sample at 1.8 × 10^9^ Ω·cm in descending order. It seems that a viologen content of 1~2 wt% greatly enhances the electric conduction of the composite, while further increasing viologen content contributes little to electric conduction, as the decreasing trend is greatly suppressed. It should be noted that the volume resistivity of the viologen/P(VDF–HFP) bulk is not comparable with traditional pure insulating polymers such as PE and PP in a 10^15^~10^16^ level. However, it could be applied with typical insulating films to form a layered structure. Thus, the typical insulating layer could withstand a high electric field, while the viologen/P(VDF–HFP) composite acts as functional layer for space charge detection. A recent investigation into Poly(3-hexylthiophene)/low-density polyethylene (P3HT/LDPE) blends indicates that a miniscule content (0.0005 wt%) of semiconducting P3HT could largely increase resistivity of the composite [36]. Thus, viologen/PVDF/P3HT ternary blends and their applications for an improved electrical insulating electrochromism system will be investigated in future works.

## 4. Conclusions

In this work, the electrochromism of a viologen/P(VDF–HFP) composite is investigated from gel to bulk structure for developing electrical insulating electrochromism material. The reversible electrochromism of viologen/P(VDF–HFP) gel is triggered by a continuous current in a range of 3~5 V in 1 s, showing quick response characteristics. This electrochromism is controlled by the reduction potential of viologen; under high voltages towards 5 V, +2 viologen can be reduced to the second stage in neutral state with a dark purple color, while under 3 V, it can only be reduced to monovalent cations in azure. In the insulating bulk structure, the electrochromism of the viologen/P(VDF–HFP) composite is triggered by space charges induced from the electrode under high electric fields larger than 10^6^ V/m. This electrochromism can be visually extended by increasing viologen content towards 5 wt%, showing a positive response to voltage amplitude and application duration. Compared with a gel structure, a viologen/P(VDF–HFP) composite bulk can withstand high electrical strength beyond 10^6^ V/m, with typical electrical insulating performance exhibiting a volume resistivity larger than 10^10^ Ω·cm. It can be further developed as an indicator for space charge accumulation in high-voltage power equipment, which exhibits potential for massive manufacturing in terms of insulation status monitoring and safety protection. Future investigations should address space charge distributions regarding easier electrochromism observations for industrial applications.

## Figures and Tables

**Table 1:** Energy structure parameters of viologen.

*λ*_g_ [nm]	*E*_red_ [V]	*E*_g_ [eV] [a]	*E*_LUMO_ [eV] [b]	*E*_HUMO_ [eV] [b]
312	−1.10, −0.72	3.97	−4.08	−8.05

(a) Energy gap was calculated from the UV–Vis absorption spectra in DMF, *E*_g_ = 1240/*λ*_g_; (b) Energy levels vs. vacuum levels were calculated from CV data along with the optically determined energy gap.

## Data Availability

Not applicable.
